# EPIYA motiefs of *cagA* and upper gastrointestinal diseases 

**Published:** 2015

**Authors:** Saeed Soleiman-Meigooni, Kaveh Baghaei, Abbas Yadegar, Samaneh Alizadeh

**Affiliations:** 1 *Infectious Diseases Research Center, University of Medical Sciences, Tehran, Iran*; 2 *Gastroenterology and Liver Diseases Research Center, Research Institute for Gastroenterology and Liver Diseases, Shahid Beheshti University of Medical Sciences, Tehran, Iran*; 3 *Shahid Beheshti University of Medical Science*

**To the Editor**

 In the April issue, *Gastroenterology and Hepatology From Bed to Bench* published a paper that focuses on the prevalence of EPIYA motiefs of *cagA* in Iranian patients and correlation with gastrointestinal disorders. Recently published research indicates that 56% of *H. pylori* isolated from Iranian patients carry *cagA*(1). It is not surprising that many *H. pylori* strains are *cagA* negative, but it should be noticed that the role of *cagA* in gastrointestinal cancers is still unclear. One of the first studies about EPIYA motifes is done by Shokrzadeh et al (2). Their study screened 190 patients, among them 141 patients (74.2%) were proven to be infected with *H. pylori* and 92 (73.6%) were positive for the *cagA* gene. To analyze the 3′-end variable region of the *cagA* gene in *H. pylori* isolated from Iranian population, they performed nucleotide sequencing of the *cagA* variable regions. Shokrzadeh et al. reported that 3' region of the *cagA* gene in Iranian strains is Western type. Although they had more samples of *cagA* positive *H. pylori* strains in comparison to that of the current study, they could not find any significant differences between EPIYA types and clinical outcomes. While the new research claim that the correlation between the presence of *H. pylori* EPIYA *cagA* motifs in the upper gastrointestinal diseases and clinical outcomes. Another study by our team (3) had similar results and could not find an association between the *cagA* status and clinical outcomes such as cancers in Iranian patients, which was in agreement with other studies in Iran (4, 5, 6,7). One more point that should be considered is about the western type of *cagA* EPIYA repeats or East Asian type repeats. The *cagA* gene is reported to be sub-typed based on the number of sequences of the repeat region in the 3′ region of the *cagA* gene (8, 9), and the *cagA* gene with multiple repeats and/or East Asian type repeats are reported to be more virulent than with fewer repeats and/or Western type repeats (8, 10). Another interesting point is about the prevalence of *cagA*, in published studies. The results showed that the prevalence of *cagA*-positive *H. pylori* in Shiraz is as high as in western countries. However, according to Baghaei et al. study we can add at this point that the reported prevalence is also similar to the eastern countries. Baghaei K et al, reported that among 231 *H.pylori*-positive patients, 154 (66.7%) patients were infected with the *cagA*-positive strains that is similar to the current studies and other countries such as Colombia, Cuba, china and neighbors of Iran, such as Turkey, Saudi and Iraq. 

Regarding the fact that Iranian *cagA* motiefs are western type, they cannot be really in related with severe gastrointestinal disorders like cancers. So it does not seem to be a reliable conclusion because of low samples size. However our previous studies with more *cagA* positive strains did not find any correlation between *cagA* repeats and cancer. Therefore, further studies will be necessary to investigate whether the *cagA* sub-typing is involved in the development of clinical outcomes among the Iranian population.
